# Technic

**Published:** 1912-03

**Authors:** John G. W. Knoll

**Affiliations:** Buffalo


### Technic.

By JOHN G. W. KNOLL, M. D., Buffalo.

NEARLY every operator has some original and many acquired methods of preparation, procedure and after treatment. Most institutions have their established technic founded and followed by the prominent and successful men on the staff. The trend of advance in surgery has been rapid and radical during the past ten years, and surgical technic has changed as often as the sun rises and sets. What we adopt as our method today, tomorrow may be discarded. Old methods again take place of the newer ones which have fallen into disrepute for some reason or other. The general trend, however, has been a decided advance particularly along the lines of thoroughness and simplicity. Any one technic is not suited for all operators.

The most judicious plan is to follow a well known and proved technic rather than to change continually to unknown and practically untried methods. The indiscriminate change of instruments, suture material, incision, procedure on viscera and closure without any other reason than to try a new fad, is unscientific, questionable and reckless. The time consumed in performing an operation depends solely upon the anatomic, surgical and pathologic knowledge of the surgeon, combined with experience and a technic which he has perfected so that not alone is he familiar with all its details, but his immediate assistants know his procedure and methods like a book.

Assistants to the operator should be so placed about the table, some on foot stools, that they are practically out of each other's way and still have a full view and convenient access to the field of operation. Two assistants are usually enough in most any operation. The first assistant's duty is to perform haemostasis, sponge, tie and cut ligatures. The second assistant's work is to see that all necessary instruments are in place and that all soiled instruments and sponges are instantly removed, also to keep the surrounding operative field free from blood as possible, by placing clean sterile towels over the soiled ones. The operator and chief assistant should never under any circumstances take their eyes off the immediate operative field, especially when infective material, pus, fluid from cysts ruptured tubes and gall bladder contents are being handled. The less time consumed in completing an operation, the more rapid is recovery from the anesthetic and shock.

Preparatory treatment to patients who are to undergo major operations in my estimation, is as essential as is the training of an athlete. Some institutions give very little preparatory treatment. A few of our most noted surgeons hold that preparatory treatment is quite unnecessary except in special cases.

The calls upon the heart, lungs, kidneys, digestive and nervous system incident to an operation, are of the most severe character. Poor results and death often follow when these important duties to the patient have been neglected.

When a person to be operated upon is taken away from the dinner table or one whose appetite was assuaged with cheese, rye bread and beer and whose gastronomic history is that of a glutton and within twenty-four hours without any other preparation than a general bath, a dose of castor oil, Tr. Iodine on the abdomen, a hysterectomy or cholecystotomy is performed; very often the undertaker is called in to confirm the prognosis. Besides a urinalysis, blood count and physical examination all patients who are to undergo an abdominal operation should come to the hospital and be placed on a limited diet and give the surgeon several days or sufficient time to note any and all pathologic conditions that may exist.

This brings to my mind a case of uterine fibroid which was to be operated. Four urinalyses were made and reported negative. The fifth specimen showed an abundant amount of sugar. The sixth specimen was again reported negative. We cannot be too careful by whom and how our examinations are conducted. The most technical skill is sometimes necessary to discover some pathologic conditions which may contra-indicate an operation. Kidney disease, myocarditis and other advanced changes in the blood vessels make operative precedure most hazardous.

While it is necessary to have sufficient and proper instruments, the average operator usually has more for his immediate use than he can possibly keep track of. How often we see the instrument tray and field of operation strewn with all kinds of knives, haemostats and scissors; the operator groveling and spending precious moments searching for this or that particular instrument. Learn to perform your operations with as few instruments as possible. It will not only save much time and eventually develop a good and rapid technic but the dangers of infection are minimized.

The question of sterilizing the hands is as simple as it is efficient: scrubbing with boiled brushes and bar soap, washing in alcohol or sterile water is all that is necessary provided that thoroughly sterile dry or wet rubber gloves are worn. Sterile trousers, shirts, apron and gown with a well fitted mask and mouth gag are the necessary garments to wear in the operating room.

The night before operation, a general bath is given and the point of selection is thoroughly scrubbed and then covered with a plain sterile gauze dressing fastened with adhesive plaster and bandage. This dressing is not removed until the patient is placed upon the operating table when the whole field and for some distance beyond is covered with Tr. Iodine and wiped with alcohol. Now the entire field is covered with large sterile towels except at the point of incision and fastened with towel clips to the skin. The entire patient from the anesthetic frame is covered with a large sterile section sheet with its slit in position.

The selection of a knife is of considerable importance, most knives have a belly, which makes it almost impossible to judge the depth of the cut. A knife with a centered point is ideal and for general work is the most satisfactory. In an abdominal operation, the incision should be carried down to the fascia at once, which is divided exposing the muscular layers, the strands of which are separated lengthwise with the handle of knife or finger. The preperitoneal fat is now separated and when haemostasis is accomplished, the peritoneum proper is picked up between forceps and cautiously divided. The incision can now be enlarged to suit the case, taking care not to injure any of the viscera.

The skin incision should be clean cut and made with one firm stroke. A fact to which I wish to call special attention is that most operators try to do their work through too small an opening. Remember that a large incision will heal just as readily and completely as a small one, in the same space of time and will give the operator better view of the anatomy and pathology to say nothing of unconstrained manipulation.

After placing a triple self-retaining retractor, the free abdominal spaces are walled off with sterile gauze towels and the flat or Trendelenburg position is assumed as the case may require. The use of many small gauze towels is somewhat annoying as the nurses are obliged to keep constant track. A better plan is to have one or two six yard towels.

The complete procedure in the removal of tubes, ovaries, uterus and appendix, likewise a resection of the stomach or bowels or operations upon the gall bladder and its ducts are not within the scope of this paper and can only be successfully performed by a skilled surgeon who has served his time as an assistant to an operator of ability.

Sutures should be prepared and handled only by persons of technical experience whose training, judgment and ability can not be questioned. The person who handles the sutures and needles must have foresight, be familiar with all operations, and to be valuable must anticipate the operator's immediate wants. The practice of changing suture nurses from time to time to give them experience is not only to be condemned from a surgical point of view but from a legal standpoint; the patient has certain rights which the surgeon must respect. It may be good practice in hospitals to give nurses instructions in preparing and handling sutures as part of their education but it is a mighty risky one to allow this continual change of persons in responsible positions in the operating room.

Closing the incision is an important part of the technic, for upon this depends the functional usefulness, appearance and final discharge of the patient from the hospital. The normal anatomic relations must be perfectly restored in situ, otherwise a most skillful operation upon the viscera will have its halo dimmed.

DRAINAGE. Upon this important part of surgical technic the life of the subject depends.

Only the most experienced surgeon can and is compelled to adopt an immediate procedure. It is a matter of vast importance and nicety of judgment to be able to decide whether or not and with what and how you shall drain. Should the case be a gangrenous appendix with a walled off, pocketed or diffused abcess or are you dealing with an ovarian abcess with mixed infection following parturition or simple gonorrhoeal pus, then and there you must without reservation decide a perfect method of drainage or closure.

The gauze or Mikulicz drain has its advantages in that if it is properly prepared and placed it will completely so to speak, dry out the pus cavity by capillary attraction from the bottom and then gradually allow the granulations and viscera to bring about a shallow and practically superficial condition. It therefore should be used in deep and extensive cavities where conditions are gangrenous and the infection mixed.

The rubber drainage tube outside of gall bladder and duct work and possibly in the cul de sac abcesses where its office is simply to carry off material favored by the forces of gravity, is not only useless but absolutely dangerous. For example: Take a gangrenous appendix with a large pus cavity. The cavity must naturally fill itself completely before the pus begins to run out of the tube. You therefore have at all times a cavity of pus in situ. Rubber tissue and combination rubber tissue and gauze or cigarette drains are principally placed in the angle of an incision where the conditions were sterile to allow superabundant serum to escape, and sometimes as a safety where infection is suspected.

The suture material used in visceral and closing up technic should be well tried and tested. Whether continous or interrupted stiches are used will depend upon the skill, judgment and experience of the operator.

The plan generally followed at the German Deaconess Hospital by the surgical staff is to use plain, fine catgut continous for the peritoneum and muscular layer; double Iodine catgut continous for fasciae, silkwormgut sub-cuticular or occasionally through and through figure of eight at angles of incision for the skin, where the structures are thick, gaping and the incision rather long.

The use of silk and linen boiled in 5% Carbolic the night before the operation and again just before using have given entirely satisfactory results in visceral and superficial work. Fine plain catgut to tie off small blood vessels and heavy Iodine for larger vessels and broad ligament work are all that is necessary.

The after treatment which essentially commences with the first dressing and which should be as simple as possible: twenty or thirty layers of plain sterile gauze after the immediate field has been wiped with Hydrogen peroxide and dried with a sterile towel and held down firmly with Z. O. adhesive plaster and then covered with several layers of absorbent cotten and bandages.

When the patient has been placed in bed, abundant warm bed clothes and hot water bottles should be placed along trunk and at the feet until circulation and respiration have become re-established.

A nurse should be in constant attendance to guard and watch the patient until normal consciousness has entirely returned.

The shock, vomiting, suffering of pain and restlessness are sequelae which follow abdominal operations. Pain is relieved with hypodermics of morphine, P. R. N. Shock, weak pulse, with camphor and oil. During the next three days, the patient will have a rather unpleasant time between pain from gas distention and surgical peritonitis. All of these disagreeable conditions can in a measure be alleviated. Allow the patient to roll gently from side to side, flex the lower limbs and occasionally brace and raise head and back with pillows. An enema of sterile salt solution and turpentine with a high rectal tube will occasionally afford much relief from pain and gas. The practice of allowing the patient to lie flat upon the back after an abdominal operation for several days is not only very uncomfortable but the accumulation of fluids in one place in the abdominal cavity is a good focus for infections and adhesions. Besides the distribution of fluids over a large surface gives the peritoneal vessels a wider area of absorption. This occasional shifting of the body from side to side also equalizes the circulation. After the effects of the anesthetic have worn off and the patient has not vomited for eight hours, weak, hot tea and small amounts of pure water can be given.

Forty-eight hours after the operation, I give Calomel gr. 1/4 each hour until eight doses have been taken and twelve hours later follow this with a normal salt enema. This is most always followed by several good sized semi-liquid stools with complete relief from pain and meteorism.

It is the duty of the surgeon and a pleasure of a conscientious one to visit his patient at least twice daily for the first four consecutive days following major surgical operations, and besides the house surgeon should see the patient morning, noon and night and any untoward complication should be reported to the surgeon in charge at once.

Specific written orders should be left with the nurse in charge whose duty it is to see that they are carried out to the letter. The most gentle and kind decorum is necessary on the part of the nurses to patient, combined with a prompt service. Any hospital or institution which cannot guarantee these services has no moral or legal right to receive patients either for surgical or medical treatment.

The duties of the hospitals do not cease in all of the above cares but they must engage the services of a competent Pathologist of unquestioned ability and whose duty it is to assist the surgeon to discover all pathologic conditions ante and post mortem in all cases which come under their observation.

Some surgeons go so far as to state that the outcome or prognosis of a surgical operation is decided upon the operating table. While this in a measure may be true I must disagree that such is not the case in all operations. I have seen a post operative case where the surgeon in charge did not know that the patient's bowels had not been moved for ten days following the operation. This patient died and whether this intestinal condition was the cause of death or not the fact remained. No effort had been made for ten days to move the bowels and all subsequent efforts were futile.

Surgical Technic is then combined knowledge of anatomy, surgical and pathologic; simple and perfected methods of procedure, absolute personal cleanliness as well, sterile instruments, suture material, operative field and dressings, associated with these, proper preparation and other treatment spell success. With these it may well be said, that cleanliness is next to Godliness.

